# Supplementary material for: Properties evaluation of silorane, low-shrinkage, non-flowable and flowable resin-based composites in dentistry
Source: PeerJ. 2015 Jun 9;3:e864. doi: 10.7717/peerj.864 (PMC4465949; doi:10.7717/peerj.864)
Supplement: Supplemental Information 1 [file peerj-03-864-s001.pdf]

Material$ into Material

Old Value	New Value	Value Label
1- Surefil flow	1	1- Surefil flow
2- Tetric flow	2	2- Tetric flow
3- Filtec flow	3	3- Filtec flow
4- Esthetic X	4	4- Esthetic X
5- TETRIC	5	5- TETRIC
6- Filtec Z 350	6	6- Filtec Z 350
7- Filtec P 90	7	7- Filtec P 90

Oneway

Descriptives

		N	Mean	Std. Deviation	Std. Error
Cont_stress	1- Surefil flow	10	2,9060	,03950	,01249
	2- Tetric flow	10	4,2170	,07602	,02404
	3- Filtec flow	10	4,1120	,05329	,01685
	4- Esthetic X	10	2,2560	,09489	,03001
	5- TETRIC	10	2,0310	,13025	,04119
	6- Filtec Z 350	10	2,1340	,07471	,02363
	7- Filtec P 90	10	1,0150	,11549	,03652
	Total	70	2,6673	1,08906	,13017
Dureza_Bottom	1- Surefil flow	10	64,8100	,04055	,01282
	2- Tetric flow	10	41,8580	,54526	,17243
	3- Filtec flow	10	45,1240	,15508	,04904
	4- Esthetic X	10	61,3210	,53494	,16916
	5- TETRIC	10	52,0290	,43514	,13760
	6- Filtec Z 350	10	63,2820	,81122	,25653
	7- Filtec P 90	10	62,3620	,69315	,21919
	Total	70	55,8266	8,80561	1,05247
Dureza_Top	1- Surefil flow	10	72,725	1,2387	,3917
	2- Tetric flow	10	55,599	,0223	,0071
	3- Filtec flow	10	53,712	1,3180	,4168
	4- Esthetic X	10	77,422	1,2494	,3951
	5- TETRIC	10	64,130	1,1496	,3635
	6- Filtec Z 350	10	78,664	,6778	,2143
	7- Filtec P 90	10	73,704	,6073	,1920
	Total	70	67,994	9,6049	1,1480
Prof_Polim	1- Surefil flow	10	3,0710	,04533	,01433
	2- Tetric flow	10	2,8930	,07025	,02221
	3- Filtec flow	10	2,8370	,13458	,04256
	4- Esthetic X	10	2,6120	,09589	,03032
	5- TETRIC	10	2,5440	,22814	,07214
	6- Filtec Z 350	10	2,5670	,13208	,04177
	7- Filtec P 90	10	2,6790	,05820	,01841
	Total	70	2,7433	,21777	,02603
Red_Cont_com	1- Surefil flow	0	.	.	.
	2- Tetric flow	0	.	.	.
	3- Filtec flow	0	.	.	.
	4- Esthetic X	10	6,2870	,12193	,03856
	5- TETRIC	10	5,8940	,23726	,07503
	6- Filtec Z 350	10	5,7400	,35978	,11377
	7- Filtec P 90	10	3,8060	,24254	,07670
	Total	40	5,4318	1,00214	,15845
Red_Cont_sem	1- Surefil flow	10	5,0590	,36892	,11666
	2- Tetric flow	0	.	.	.
	3- Filtec flow	0	.	.	.
	4- Esthetic X	10	8,5210	,39051	,12349
	5- TETRIC	10	8,4950	,49437	,15633
	6- Filtec Z 350	10	8,4070	,46636	,14748
	7- Filtec P 90	10	4,8730	,37665	,11911
	Total	50	7,0710	1,78415	,25232

Descriptives

		95% Confidence Interval for Mean		Minimum	Maximum
		Lower Bound	Upper Bound		
Cont_stress	1- Surefil flow	2,8777	2,9343	2,82	2,97
	2- Tetric flow	4,1626	4,2714	4,07	4,33
	3- Filtec flow	4,0739	4,1501	4,01	4,19
	4- Esthetic X	2,1881	2,3239	2,13	2,41
	5- TETRIC	1,9378	2,1242	1,82	2,30
	6- Filtec Z 350	2,0806	2,1874	1,99	2,25
	7- Filtec P 90	,9324	1,0976	,86	1,20
	Total	2,4076	2,9270	,86	4,33
Dureza_Bottom	1- Surefil flow	64,7810	64,8390	64,73	64,87
	2- Tetric flow	41,4679	42,2481	40,87	42,93
	3- Filtec flow	45,0131	45,2349	44,86	45,34
	4- Esthetic X	60,9383	61,7037	60,38	62,22
	5- TETRIC	51,7177	52,3403	51,16	52,84
	6- Filtec Z 350	62,7017	63,8623	61,53	64,87
	7- Filtec P 90	61,8661	62,8579	61,07	63,73
	Total	53,7269	57,9262	40,87	64,87
Dureza_Top	1- Surefil flow	71,839	73,611	70,5	74,1
	2- Tetric flow	55,583	55,615	55,6	55,6
	3- Filtec flow	52,769	54,655	50,9	55,1
	4- Esthetic X	76,528	78,316	76,2	79,0
	5- TETRIC	63,308	64,952	61,9	65,9
	6- Filtec Z 350	78,179	79,149	77,7	80,1
	7- Filtec P 90	73,270	74,138	72,2	74,6
	Total	65,704	70,284	50,9	80,1
Prof_Polim	1- Surefil flow	3,0386	3,1034	2,99	3,14
	2- Tetric flow	2,8427	2,9433	2,79	2,99
	3- Filtec flow	2,7407	2,9333	2,59	3,03
	4- Esthetic X	2,5434	2,6806	2,37	2,73
	5- TETRIC	2,3808	2,7072	2,20	2,86
	6- Filtec Z 350	2,4725	2,6615	2,35	2,77
	7- Filtec P 90	2,6374	2,7206	2,59	2,77
	Total	2,6914	2,7952	2,20	3,14
Red_Cont_com	1- Surefil flow	.	.	.	.
	2- Tetric flow	.	.	.	.
	3- Filtec flow	.	.	.	.
	4- Esthetic X	6,1998	6,3742	6,01	6,47
	5- TETRIC	5,7243	6,0637	5,60	6,28
	6- Filtec Z 350	5,4826	5,9974	5,27	6,41
	7- Filtec P 90	3,6325	3,9795	3,32	4,32
	Total	5,1113	5,7522	3,32	6,47
Red_Cont_sem	1- Surefil flow	4,7951	5,3229	4,55	5,54
	2- Tetric flow	.	.	.	.
	3- Filtec flow	.	.	.	.
	4- Esthetic X	8,2416	8,8004	7,84	9,38
	5- TETRIC	8,1413	8,8487	7,70	9,38
	6- Filtec Z 350	8,0734	8,7406	7,48	9,16
	7- Filtec P 90	4,6036	5,1424	4,14	5,66
	Total	6,5640	7,5780	4,14	9,38

ANOVA

		Sum of Squares	df	Mean Square	F	Sig.
Cont_stress	Between Groups	81,343	6	13,557	1723,255	,000
	Within Groups	,496	63	,008		
	Total	81,838	69			
Dureza_Bottom	Between Groups	5332,735	6	888,789	3211,846	,000
	Within Groups	17,433	63	,277		
	Total	5350,169	69			
Dureza_Top	Between Groups	6302,646	6	1050,441	1053,063	,000
	Within Groups	62,843	63	,998		
	Total	6365,489	69			
Prof_Polim	Between Groups	2,308	6	,385	25,118	,000
	Within Groups	,965	63	,015		
	Total	3,272	69			
Red_Cont_com	Between Groups	36,832	3	12,277	189,296	,000
	Within Groups	2,335	36	,065		
	Total	39,167	39			
Red_Cont_sem	Between Groups	147,945	4	36,986	207,238	,000
	Within Groups	8,031	45	,178		
	Total	155,976	49			

Post Hoc Tests

Homogeneous Subsets

Cont_stress

	Material	N	Subset for alpha = .05			
			1	2	3	4
Student-Newman-Keuls[a]	7- Filtec P 90	10	1,0150			
	5- TETRIC	10		2,0310		
	6- Filtec Z 350	10			2,1340	
	4- Esthetic X	10				2,2560
	1- Surefil flow	10				
	3- Filtec flow	10				
	2- Tetric flow	10				
	Sig.		1,000	1,000	1,000	1,000
Tukey HSD[a]	7- Filtec P 90	10	1,0150			
	5- TETRIC	10		2,0310		
	6- Filtec Z 350	10		2,1340		
	4- Esthetic X	10			2,2560	
	1- Surefil flow	10				2,9060
	3- Filtec flow	10				
	2- Tetric flow	10				
	Sig.		1,000	,145	1,000	1,000
Tukey B[a]	7- Filtec P 90	10	1,0150			
	5- TETRIC	10		2,0310		
	6- Filtec Z 350	10			2,1340	
	4- Esthetic X	10				2,2560
	1- Surefil flow	10				
	3- Filtec flow	10				
	2- Tetric flow	10				
Duncan[a]	7- Filtec P 90	10	1,0150			
	5- TETRIC	10		2,0310		
	6- Filtec Z 350	10			2,1340	
	4- Esthetic X	10				2,2560
	1- Surefil flow	10				
	3- Filtec flow	10				
	2- Tetric flow	10				
	Sig.		1,000	1,000	1,000	1,000
Scheffe[a]	7- Filtec P 90	10	1,0150			
	5- TETRIC	10		2,0310		
	6- Filtec Z 350	10		2,1340	2,1340	
	4- Esthetic X	10			2,2560	
	1- Surefil flow	10				2,9060
	3- Filtec flow	10				
	2- Tetric flow	10				
	Sig.		1,000	,359	,169	1,000
Gabriel[a]	7- Filtec P 90	10	1,0150			
	5- TETRIC	10		2,0310		
	6- Filtec Z 350	10		2,1340	2,1340	
	4- Esthetic X	10			2,2560	
	1- Surefil flow	10				2,9060
	3- Filtec flow	10				
	2- Tetric flow	10				
	Sig.		1,000	,209	,062	1,000

Means for groups in homogeneous subsets are displayed.

Cont_stress

	Material	N	Subset for alpha = .05			
			1	2	3	4
Ryan-Einot-Gabriel-Welsch F	7- Filtec P 90	10	1,0150			
	5- TETRIC	10		2,0310		
	6- Filtec Z 350	10			2,1340	
	4- Esthetic X	10				2,2560
	1- Surefil flow	10				
	3- Filtec flow	10				
	2- Tetric flow	10				
	Sig.		1,000	1,000	1,000	1,000
Ryan-Einot-Gabriel-Welsch Range[b]	7- Filtec P 90	10	1,0150			
	5- TETRIC	10		2,0310		
	6- Filtec Z 350	10			2,1340	
	4- Esthetic X	10				2,2560
	1- Surefil flow	10				
	3- Filtec flow	10				
	2- Tetric flow	10				
	Sig.		1,000	1,000	1,000	1,000
Hochberg[a]	7- Filtec P 90	10	1,0150			
	5- TETRIC	10		2,0310		
	6- Filtec Z 350	10		2,1340	2,1340	
	4- Esthetic X	10			2,2560	
	1- Surefil flow	10				2,9060
	3- Filtec flow	10				
	2- Tetric flow	10				
	Sig.		1,000	,209	,062	1,000

Means for groups in homogeneous subsets are displayed.

Cont_stress

	Material	Subset for alpha = .05		
		5	6	7
Student-Newman-Keuls[a]	7- Filtec P 90			
	5- TETRIC			
	6- Filtec Z 350			
	4- Esthetic X			
	1- Surefil flow	2,9060		
	3- Filtec flow		4,1120	
	2- Tetric flow			4,2170
	Sig.	1,000	1,000	1,000
Tukey HSD[a]	7- Filtec P 90			
	5- TETRIC			
	6- Filtec Z 350			
	4- Esthetic X			
	1- Surefil flow			
	3- Filtec flow	4,1120		
	2- Tetric flow	4,2170		
	Sig.	,130		
Tukey B[a]	7- Filtec P 90			
	5- TETRIC			
	6- Filtec Z 350			
	4- Esthetic X			
	1- Surefil flow	2,9060		
	3- Filtec flow		4,1120	
	2- Tetric flow			4,2170
Duncan[a]	7- Filtec P 90			
	5- TETRIC			
	6- Filtec Z 350			
	4- Esthetic X			
	1- Surefil flow	2,9060		
	3- Filtec flow		4,1120	
	2- Tetric flow			4,2170
	Sig.	1,000	1,000	1,000
Scheffe[a]	7- Filtec P 90			
	5- TETRIC			
	6- Filtec Z 350			
	4- Esthetic X			
	1- Surefil flow			
	3- Filtec flow	4,1120		
	2- Tetric flow	4,2170		
	Sig.	,335		
Gabriel[a]	7- Filtec P 90			
	5- TETRIC			
	6- Filtec Z 350			
	4- Esthetic X			
	1- Surefil flow			
	3- Filtec flow	4,1120		
	2- Tetric flow	4,2170		
	Sig.	,186		

Means for groups in homogeneous subsets are displayed.

Cont_stress

	Material	Subset for alpha = .05		
		5	6	7
Ryan-Einot-Gabriel-Welsch F	7- Filtec P 90			
	5- TETRIC			
	6- Filtec Z 350			
	4- Esthetic X			
	1- Surefil flow	2,9060		
	3- Filtec flow		4,1120	
	2- Tetric flow			4,2170
	Sig.	1,000	1,000	1,000
Ryan-Einot-Gabriel-Welsch Range[b]	7- Filtec P 90			
	5- TETRIC			
	6- Filtec Z 350			
	4- Esthetic X			
	1- Surefil flow	2,9060		
	3- Filtec flow		4,1120	
	2- Tetric flow			4,2170
	Sig.	1,000	1,000	1,000
Hochberg[a]	7- Filtec P 90			
	5- TETRIC			
	6- Filtec Z 350			
	4- Esthetic X			
	1- Surefil flow			
	3- Filtec flow	4,1120		
	2- Tetric flow	4,2170		
	Sig.	,186		

Means for groups in homogeneous subsets are displayed.

a. Uses Harmonic Mean Sample Size = 10,000.

b. Critical values are not monotonic for these data. Substitutions have been made to ensure monotonicity. Type I error is therefore smaller.

Dureza_Bottom

	Material	N	Subset for alpha = .05			
			1	2	3	4
Student-Newman-Keuls[a]	2- Tetric flow	10	41,8580			
	3- Filtec flow	10		45,1240		
	5- TETRIC	10			52,0290	
	4- Esthetic X	10				61,3210
	7- Filtec P 90	10				
	6- Filtec Z 350	10				
	1- Surefil flow	10				
	Sig.		1,000	1,000	1,000	1,000
Tukey HSD[a]	2- Tetric flow	10	41,8580			
	3- Filtec flow	10		45,1240		
	5- TETRIC	10			52,0290	
	4- Esthetic X	10				61,3210
	7- Filtec P 90	10				
	6- Filtec Z 350	10				
	1- Surefil flow	10				
	Sig.		1,000	1,000	1,000	1,000
Tukey B[a]	2- Tetric flow	10	41,8580			
	3- Filtec flow	10		45,1240		
	5- TETRIC	10			52,0290	
	4- Esthetic X	10				61,3210
	7- Filtec P 90	10				
	6- Filtec Z 350	10				
	1- Surefil flow	10				
Duncan[a]	2- Tetric flow	10	41,8580			
	3- Filtec flow	10		45,1240		
	5- TETRIC	10			52,0290	
	4- Esthetic X	10				61,3210
	7- Filtec P 90	10				
	6- Filtec Z 350	10				
	1- Surefil flow	10				
	Sig.		1,000	1,000	1,000	1,000
Scheffe[a]	2- Tetric flow	10	41,8580			
	3- Filtec flow	10		45,1240		
	5- TETRIC	10			52,0290	
	4- Esthetic X	10				61,3210
	7- Filtec P 90	10				
	6- Filtec Z 350	10				
	1- Surefil flow	10				
	Sig.		1,000	1,000	1,000	1,000
Gabriel[a]	2- Tetric flow	10	41,8580			
	3- Filtec flow	10		45,1240		
	5- TETRIC	10			52,0290	
	4- Esthetic X	10				61,3210
	7- Filtec P 90	10				
	6- Filtec Z 350	10				
	1- Surefil flow	10				
	Sig.		1,000	1,000	1,000	1,000

Means for groups in homogeneous subsets are displayed.

Dureza_Bottom

	Material	N	Subset for alpha = .05			
			1	2	3	4
Ryan-Einot-Gabriel-Welsch F	2- Tetric flow	10	41,8580			
	3- Filtec flow	10		45,1240		
	5- TETRIC	10			52,0290	
	4- Esthetic X	10				61,3210
	7- Filtec P 90	10				
	6- Filtec Z 350	10				
	1- Surefil flow	10				
	Sig.		1,000	1,000	1,000	1,000
Ryan-Einot-Gabriel-Welsch Range[b]	2- Tetric flow	10	41,8580			
	3- Filtec flow	10		45,1240		
	5- TETRIC	10			52,0290	
	4- Esthetic X	10				61,3210
	7- Filtec P 90	10				
	6- Filtec Z 350	10				
	1- Surefil flow	10				
	Sig.		1,000	1,000	1,000	1,000
Hochberg[a]	2- Tetric flow	10	41,8580			
	3- Filtec flow	10		45,1240		
	5- TETRIC	10			52,0290	
	4- Esthetic X	10				61,3210
	7- Filtec P 90	10				
	6- Filtec Z 350	10				
	1- Surefil flow	10				
	Sig.		1,000	1,000	1,000	1,000

Means for groups in homogeneous subsets are displayed.

Dureza_Bottom

	Material	Subset for alpha = .05		
		5	6	7
Student-Newman-Keuls[a]	2- Tetric flow			
	3- Filtec flow			
	5- TETRIC			
	4- Esthetic X			
	7- Filtec P 90	62,3620		
	6- Filtec Z 350		63,2820	
	1- Surefil flow			64,8100
	Sig.	1,000	1,000	1,000
Tukey HSD[a]	2- Tetric flow			
	3- Filtec flow			
	5- TETRIC			
	4- Esthetic X			
	7- Filtec P 90	62,3620		
	6- Filtec Z 350		63,2820	
	1- Surefil flow			64,8100
	Sig.	1,000	1,000	1,000
Tukey B[a]	2- Tetric flow			
	3- Filtec flow			
	5- TETRIC			
	4- Esthetic X			
	7- Filtec P 90	62,3620		
	6- Filtec Z 350		63,2820	
	1- Surefil flow			64,8100
Duncan[a]	2- Tetric flow			
	3- Filtec flow			
	5- TETRIC			
	4- Esthetic X			
	7- Filtec P 90	62,3620		
	6- Filtec Z 350		63,2820	
	1- Surefil flow			64,8100
	Sig.	1,000	1,000	1,000
Scheffe[a]	2- Tetric flow			
	3- Filtec flow			
	5- TETRIC			
	4- Esthetic X			
	7- Filtec P 90	62,3620		
	6- Filtec Z 350		63,2820	
	1- Surefil flow			64,8100
	Sig.	1,000	1,000	1,000
Gabriel[a]	2- Tetric flow			
	3- Filtec flow			
	5- TETRIC			
	4- Esthetic X			
	7- Filtec P 90	62,3620		
	6- Filtec Z 350		63,2820	
	1- Surefil flow			64,8100
	Sig.	1,000	1,000	1,000

Means for groups in homogeneous subsets are displayed.

Dureza_Bottom

	Material	Subset for alpha = .05		
		5	6	7
Ryan-Einot-Gabriel-Welsch F	2- Tetric flow			
	3- Filtec flow			
	5- TETRIC			
	4- Esthetic X			
	7- Filtec P 90	62,3620		
	6- Filtec Z 350		63,2820	
	1- Surefil flow			64,8100
	Sig.	1,000	1,000	1,000
Ryan-Einot-Gabriel-Welsch Range[b]	2- Tetric flow			
	3- Filtec flow			
	5- TETRIC			
	4- Esthetic X			
	7- Filtec P 90	62,3620		
	6- Filtec Z 350		63,2820	
	1- Surefil flow			64,8100
	Sig.	1,000	1,000	1,000
Hochberg[a]	2- Tetric flow			
	3- Filtec flow			
	5- TETRIC			
	4- Esthetic X			
	7- Filtec P 90	62,3620		
	6- Filtec Z 350		63,2820	
	1- Surefil flow			64,8100
	Sig.	1,000	1,000	1,000

Means for groups in homogeneous subsets are displayed.

a. Uses Harmonic Mean Sample Size = 10,000.

b. Critical values are not monotonic for these data. Substitutions have been made to ensure monotonicity. Type I error is therefore smaller.

Dureza_Top

	Material	N	Subset for alpha = .05			
			1	2	3	4
Student-Newman-Keuls[a]	3- Filtec flow	10	53,712			
	2- Tetric flow	10		55,599		
	5- TETRIC	10			64,130	
	1- Surefil flow	10				72,725
	7- Filtec P 90	10				
	4- Esthetic X	10				
	6- Filtec Z 350	10				
	Sig.		1,000	1,000	1,000	1,000
Tukey HSD[a]	3- Filtec flow	10	53,712			
	2- Tetric flow	10		55,599		
	5- TETRIC	10			64,130	
	1- Surefil flow	10				72,725
	7- Filtec P 90	10				73,704
	4- Esthetic X	10				
	6- Filtec Z 350	10				
	Sig.		1,000	1,000	1,000	,315
Tukey B[a]	3- Filtec flow	10	53,712			
	2- Tetric flow	10		55,599		
	5- TETRIC	10			64,130	
	1- Surefil flow	10				72,725
	7- Filtec P 90	10				73,704
	4- Esthetic X	10				
	6- Filtec Z 350	10				
Duncan[a]	3- Filtec flow	10	53,712			
	2- Tetric flow	10		55,599		
	5- TETRIC	10			64,130	
	1- Surefil flow	10				72,725
	7- Filtec P 90	10				
	4- Esthetic X	10				
	6- Filtec Z 350	10				
	Sig.		1,000	1,000	1,000	1,000
Scheffe[a]	3- Filtec flow	10	53,712			
	2- Tetric flow	10		55,599		
	5- TETRIC	10			64,130	
	1- Surefil flow	10				72,725
	7- Filtec P 90	10				73,704
	4- Esthetic X	10				
	6- Filtec Z 350	10				
	Sig.		1,000	1,000	1,000	,573
Gabriel[a]	3- Filtec flow	10	53,712			
	2- Tetric flow	10		55,599		
	5- TETRIC	10			64,130	
	1- Surefil flow	10				72,725
	7- Filtec P 90	10				73,704
	4- Esthetic X	10				
	6- Filtec Z 350	10				
	Sig.		1,000	1,000	1,000	,470

Means for groups in homogeneous subsets are displayed.

Dureza_Top

	Material	N	Subset for alpha = .05			
			1	2	3	4
Ryan-Einot-Gabriel-Welsch F	3- Filtec flow	10	53,712			
	2- Tetric flow	10		55,599		
	5- TETRIC	10			64,130	
	1- Surefil flow	10				72,725
	7- Filtec P 90	10				73,704
	4- Esthetic X	10				
	6- Filtec Z 350	10				
	Sig.		1,000	1,000	1,000	,108
Ryan-Einot-Gabriel-Welsch Range[b]	3- Filtec flow	10	53,712			
	2- Tetric flow	10		55,599		
	5- TETRIC	10			64,130	
	1- Surefil flow	10				72,725
	7- Filtec P 90	10				73,704
	4- Esthetic X	10				
	6- Filtec Z 350	10				
	Sig.		1,000	1,000	1,000	,108
Hochberg[a]	3- Filtec flow	10	53,712			
	2- Tetric flow	10		55,599		
	5- TETRIC	10			64,130	
	1- Surefil flow	10				72,725
	7- Filtec P 90	10				73,704
	4- Esthetic X	10				
	6- Filtec Z 350	10				
	Sig.		1,000	1,000	1,000	,470

Means for groups in homogeneous subsets are displayed.

Dureza_Top

	Material	Subset for alpha = .05		
		5	6	7
Student-Newman-Keuls[a]	3- Filtec flow			
	2- Tetric flow			
	5- TETRIC			
	1- Surefil flow			
	7- Filtec P 90	73,704		
	4- Esthetic X		77,422	
	6- Filtec Z 350			78,664
	Sig.	1,000	1,000	1,000
Tukey HSD[a]	3- Filtec flow			
	2- Tetric flow			
	5- TETRIC			
	1- Surefil flow			
	7- Filtec P 90			
	4- Esthetic X	77,422		
	6- Filtec Z 350	78,664		
	Sig.	,096		
Tukey B[a]	3- Filtec flow			
	2- Tetric flow			
	5- TETRIC			
	1- Surefil flow			
	7- Filtec P 90			
	4- Esthetic X	77,422		
	6- Filtec Z 350		78,664	
Duncan[a]	3- Filtec flow			
	2- Tetric flow			
	5- TETRIC			
	1- Surefil flow			
	7- Filtec P 90	73,704		
	4- Esthetic X		77,422	
	6- Filtec Z 350			78,664
	Sig.	1,000	1,000	1,000
Scheffe[a]	3- Filtec flow			
	2- Tetric flow			
	5- TETRIC			
	1- Surefil flow			
	7- Filtec P 90			
	4- Esthetic X	77,422		
	6- Filtec Z 350	78,664		
	Sig.	,275		
Gabriel[a]	3- Filtec flow			
	2- Tetric flow			
	5- TETRIC			
	1- Surefil flow			
	7- Filtec P 90			
	4- Esthetic X	77,422		
	6- Filtec Z 350	78,664		
	Sig.	,135		

Means for groups in homogeneous subsets are displayed.

Dureza_Top

	Material	Subset for alpha = .05		
		5	6	7
Ryan-Einot-Gabriel-Welsch F	3- Filtec flow			
	2- Tetric flow			
	5- TETRIC			
	1- Surefil flow			
	7- Filtec P 90			
	4- Esthetic X	77,422		
	6- Filtec Z 350		78,664	
	Sig.	1,000	1,000	
Ryan-Einot-Gabriel-Welsch Range[b]	3- Filtec flow			
	2- Tetric flow			
	5- TETRIC			
	1- Surefil flow			
	7- Filtec P 90			
	4- Esthetic X	77,422		
	6- Filtec Z 350		78,664	
	Sig.	1,000	1,000	
Hochberg[a]	3- Filtec flow			
	2- Tetric flow			
	5- TETRIC			
	1- Surefil flow			
	7- Filtec P 90			
	4- Esthetic X	77,422		
	6- Filtec Z 350	78,664		
	Sig.	,135		

Means for groups in homogeneous subsets are displayed.

a. Uses Harmonic Mean Sample Size = 10,000.

b. Critical values are not monotonic for these data. Substitutions have been made to ensure monotonicity. Type I error is therefore smaller.

Prof_Polim

	Material	N	Subset for alpha = .05			
			1	2	3	4
Student-Newman-Keuls[a]	5- TETRIC	10	2,5440			
	6- Filtec Z 350	10	2,5670			
	4- Esthetic X	10	2,6120			
	7- Filtec P 90	10	2,6790			
	3- Filtec flow	10		2,8370		
	2- Tetric flow	10		2,8930		
	1- Surefil flow	10			3,0710	
	Sig.		,080	,315	1,000	
Tukey HSD[a]	5- TETRIC	10	2,5440			
	6- Filtec Z 350	10	2,5670			
	4- Esthetic X	10	2,6120			
	7- Filtec P 90	10	2,6790	2,6790		
	3- Filtec flow	10		2,8370	2,8370	
	2- Tetric flow	10			2,8930	
	1- Surefil flow	10				3,0710
	Sig.		,200	,080	,949	1,000

Means for groups in homogeneous subsets are displayed.

Prof_Polim

	Material	N	Subset for alpha = .05			
			1	2	3	4
Tukey B[a]	5- TETRIC	10	2,5440			
	6- Filtec Z 350	10	2,5670			
	4- Esthetic X	10	2,6120			
	7- Filtec P 90	10	2,6790			
	3- Filtec flow	10		2,8370		
	2- Tetric flow	10		2,8930		
	1- Surefil flow	10			3,0710	
Duncan[a]	5- TETRIC	10	2,5440			
	6- Filtec Z 350	10	2,5670	2,5670		
	4- Esthetic X	10	2,6120	2,6120		
	7- Filtec P 90	10		2,6790		
	3- Filtec flow	10			2,8370	
	2- Tetric flow	10			2,8930	
	1- Surefil flow	10				3,0710
	Sig.		,252	,059	,315	1,000
Scheffe[a]	5- TETRIC	10	2,5440			
	6- Filtec Z 350	10	2,5670			
	4- Esthetic X	10	2,6120			
	7- Filtec P 90	10	2,6790	2,6790		
	3- Filtec flow	10		2,8370	2,8370	
	2- Tetric flow	10			2,8930	2,8930
	1- Surefil flow	10				3,0710
	Sig.		,439	,245	,984	,130
Gabriel[a]	5- TETRIC	10	2,5440			
	6- Filtec Z 350	10	2,5670			
	4- Esthetic X	10	2,6120			
	7- Filtec P 90	10	2,6790	2,6790		
	3- Filtec flow	10		2,8370	2,8370	
	2- Tetric flow	10			2,8930	
	1- Surefil flow	10				3,0710
	Sig.		,295	,111	,999	1,000
Ryan-Einot-Gabriel-Welsch F	5- TETRIC	10	2,5440			
	6- Filtec Z 350	10	2,5670			
	4- Esthetic X	10	2,6120			
	7- Filtec P 90	10	2,6790			
	3- Filtec flow	10		2,8370		
	2- Tetric flow	10		2,8930		
	1- Surefil flow	10			3,0710	
	Sig.		,144	,735	1,000	
Ryan-Einot-Gabriel-Welsch Range[b]	5- TETRIC	10	2,5440			
	6- Filtec Z 350	10	2,5670			
	4- Esthetic X	10	2,6120			
	7- Filtec P 90	10	2,6790			
	3- Filtec flow	10		2,8370		
	2- Tetric flow	10		2,8930		
	1- Surefil flow	10			3,0710	
	Sig.		,136	,735	1,000	

Means for groups in homogeneous subsets are displayed.

Prof_Polim

	Material	N	Subset for alpha = .05			
			1	2	3	4
Hochberg[a]	5- TETRIC	10	2,5440			
	6- Filtec Z 350	10	2,5670			
	4- Esthetic X	10	2,6120			
	7- Filtec P 90	10	2,6790	2,6790		
	3- Filtec flow	10		2,8370	2,8370	
	2- Tetric flow	10			2,8930	
	1- Surefil flow	10				3,0710
	Sig.		,295	,111	,999	1,000

Means for groups in homogeneous subsets are displayed.

a. Uses Harmonic Mean Sample Size = 10,000.

b. Critical values are not monotonic for these data. Substitutions have been made to ensure monotonicity. Type I error is therefore smaller.

Oneway

Descriptives

Red_Cont_com

	N	Mean	Std. Deviation	Std. Error	95% Confidence Interval for Mean	
					Lower Bound	Upper Bound
4- Esthetic X	10	6,2870	,12193	,03856	6,1998	6,3742
5- TETRIC	10	5,8940	,23726	,07503	5,7243	6,0637
6- Filtec Z 350	10	5,7400	,35978	,11377	5,4826	5,9974
7- Filtec P 90	10	3,8060	,24254	,07670	3,6325	3,9795
Total	40	5,4318	1,00214	,15845	5,1113	5,7522

Descriptives

Red_Cont_com

	Minimum	Maximum
4- Esthetic X	6,01	6,47
5- TETRIC	5,60	6,28
6- Filtec Z 350	5,27	6,41
7- Filtec P 90	3,32	4,32
Total	3,32	6,47

ANOVA

Red_Cont_com

	Sum of Squares	df	Mean Square	F	Sig.
Between Groups	36,832	3	12,277	189,296	,000
Within Groups	2,335	36	,065		
Total	39,167	39			

Post Hoc Tests

Homogeneous Subsets

Red_Cont_com

	Material	N	Subset for alpha = .05		
			1	2	3
Student-Newman-Keuls[a]	7- Filtec P 90	10	3,8060		
	6- Filtec Z 350	10		5,7400	
	5- TETRIC	10		5,8940	
	4- Esthetic X	10			6,2870
	Sig.		1,000	,185	1,000
Tukey HSD[a]	7- Filtec P 90	10	3,8060		
	6- Filtec Z 350	10		5,7400	
	5- TETRIC	10		5,8940	
	4- Esthetic X	10			6,2870
	Sig.		1,000	,537	1,000
Tukey B[a]	7- Filtec P 90	10	3,8060		
	6- Filtec Z 350	10		5,7400	
	5- TETRIC	10		5,8940	
	4- Esthetic X	10			6,2870
Duncan[a]	7- Filtec P 90	10	3,8060		
	6- Filtec Z 350	10		5,7400	
	5- TETRIC	10		5,8940	
	4- Esthetic X	10			6,2870
	Sig.		1,000	,185	1,000
Scheffe[a]	7- Filtec P 90	10	3,8060		
	6- Filtec Z 350	10		5,7400	
	5- TETRIC	10		5,8940	
	4- Esthetic X	10			6,2870
	Sig.		1,000	,613	1,000
Gabriel[a]	7- Filtec P 90	10	3,8060		
	6- Filtec Z 350	10		5,7400	
	5- TETRIC	10		5,8940	
	4- Esthetic X	10			6,2870
	Sig.		1,000	,689	1,000
Ryan-Einot-Gabriel-Welsch F	7- Filtec P 90	10	3,8060		
	6- Filtec Z 350	10		5,7400	
	5- TETRIC	10		5,8940	
	4- Esthetic X	10			6,2870
	Sig.		1,000	,335	1,000
Ryan-Einot-Gabriel-Welsch Range[b]	7- Filtec P 90	10	3,8060		
	6- Filtec Z 350	10		5,7400	
	5- TETRIC	10		5,8940	
	4- Esthetic X	10			6,2870
	Sig.		1,000	,335	1,000
Hochberg[a]	7- Filtec P 90	10	3,8060		
	6- Filtec Z 350	10		5,7400	
	5- TETRIC	10		5,8940	
	4- Esthetic X	10			6,2870
	Sig.		1,000	,689	1,000

Means for groups in homogeneous subsets are displayed.

a. Uses Harmonic Mean Sample Size = 10,000.

b. Critical values are not monotonic for these data. Substitutions have been made to ensure monotonicity. Type I error is therefore smaller.

Oneway

Descriptives

Red_Cont_sem

	N	Mean	Std. Deviation	Std. Error	95% Confidence Interval for Mean	
					Lower Bound	Upper Bound
1- Surefil flow	10	5,0590	,36892	,11666	4,7951	5,3229
4- Esthetic X	10	8,5210	,39051	,12349	8,2416	8,8004
5- TETRIC	10	8,4950	,49437	,15633	8,1413	8,8487
6- Filtec Z 350	10	8,4070	,46636	,14748	8,0734	8,7406
7- Filtec P 90	10	4,8730	,37665	,11911	4,6036	5,1424
Total	50	7,0710	1,78415	,25232	6,5640	7,5780

Descriptives

Red_Cont_sem

	Minimum	Maximum
1- Surefil flow	4,55	5,54
4- Esthetic X	7,84	9,38
5- TETRIC	7,70	9,38
6- Filtec Z 350	7,48	9,16
7- Filtec P 90	4,14	5,66
Total	4,14	9,38

ANOVA

Red_Cont_sem

	Sum of Squares	df	Mean Square	F	Sig.
Between Groups	147,945	4	36,986	207,238	,000
Within Groups	8,031	45	,178		
Total	155,976	49			

Post Hoc Tests

Homogeneous Subsets

Red_Cont_sem

	Material	N	Subset for alpha = .05	
			1	2
Student-Newman-Keuls[a]	7- Filtec P 90	10	4,8730	
	1- Surefil flow	10	5,0590	
	6- Filtec Z 350	10		8,4070
	5- TETRIC	10		8,4950
	4- Esthetic X	10		8,5210
	Sig.		,330	,819
Tukey HSD[a]	7- Filtec P 90	10	4,8730	
	1- Surefil flow	10	5,0590	
	6- Filtec Z 350	10		8,4070
	5- TETRIC	10		8,4950
	4- Esthetic X	10		8,5210
	Sig.		,861	,974

Means for groups in homogeneous subsets are displayed.

Red_Cont_sem

	Material	N	Subset for alpha = .05	
			1	2
Tukey B[a]	7- Filtec P 90	10	4,8730	
	1- Surefil flow	10	5,0590	
	6- Filtec Z 350	10		8,4070
	5- TETRIC	10		8,4950
	4- Esthetic X	10		8,5210
Duncan[a]	7- Filtec P 90	10	4,8730	
	1- Surefil flow	10	5,0590	
	6- Filtec Z 350	10		8,4070
	5- TETRIC	10		8,4950
	4- Esthetic X	10		8,5210
	Sig.		,330	,575
Scheffe[a]	7- Filtec P 90	10	4,8730	
	1- Surefil flow	10	5,0590	
	6- Filtec Z 350	10		8,4070
	5- TETRIC	10		8,4950
	4- Esthetic X	10		8,5210
	Sig.		,913	,985
Gabriel[a]	7- Filtec P 90	10	4,8730	
	1- Surefil flow	10	5,0590	
	6- Filtec Z 350	10		8,4070
	5- TETRIC	10		8,4950
	4- Esthetic X	10		8,5210
	Sig.		,977	1,000
Ryan-Einot-Gabriel-Welsch F	7- Filtec P 90	10	4,8730	
	1- Surefil flow	10	5,0590	
	6- Filtec Z 350	10		8,4070
	5- TETRIC	10		8,4950
	4- Esthetic X	10		8,5210
	Sig.		,633	,942
Ryan-Einot-Gabriel-Welsch Range[b]	7- Filtec P 90	10	4,8730	
	1- Surefil flow	10	5,0590	
	6- Filtec Z 350	10		8,4070
	5- TETRIC	10		8,4950
	4- Esthetic X	10		8,5210
	Sig.		,633	,942
Hochberg[a]	7- Filtec P 90	10	4,8730	
	1- Surefil flow	10	5,0590	
	6- Filtec Z 350	10		8,4070
	5- TETRIC	10		8,4950
	4- Esthetic X	10		8,5210
	Sig.		,977	1,000

Means for groups in homogeneous subsets are displayed.

a. Uses Harmonic Mean Sample Size = 10,000.

b. Critical values are not monotonic for these data. Substitutions have been made to ensure monotonicity. Type I error is therefore smaller.
